# Synthesis, Chemosensory Properties, and Self-Assembly of Terpyridine-Containing Conjugated Polycarbazole through RAFT Polymerization and Heck Coupling Reaction

**DOI:** 10.3390/polym9090427

**Published:** 2017-09-07

**Authors:** Po-Chih Yang, Si-Qiao Li, Yueh-Han Chien, Ta-Lun Tao, Ruo-Yun Huang, Hsueh-Yu Chen

**Affiliations:** Department of Chemical Engineering and Materials Science, Yuan Ze University, Chung-Li, Taoyuan City 32003, Taiwan; s1025242@mail.yzu.edu.tw (S.-Q.L.); john7777777a@gmail.com (Y.-H.C.); s1031032@mail.yzu.edu.tw (T.-L.T.); s1031050@mail.yzu.edu.tw (R.-Y.H.); s1031104@mail.yzu.edu.tw (H.-Y.C.)

**Keywords:** carbazole, reversible addition-fragmentation transfer (RAFT), Heck reaction, sensing

## Abstract

We report the responsive fluorescence chemosensory phenomena of a carbazole-functionalized crosslinked polymer (PCaT) with pendent terpyridine (tpy) groups as receptors of metal ions. The polymer was synthesized using Heck polymerization between 3,6-dibromide groups in a carbazole-based polymer (PC2Br) and divinyl tpy monomer. The effects of the polymeric structure on the optical and chemosensory properties of the PCaT were compared with those of a carbazole-tpy alternating conjugated polymer (PCT). Photoluminescence titrations demonstrated that the PCaT and PCT had the high sensing ability toward Fe^3+^ ions, with Stern–Volmer constants of 8.10 × 10^4^ and 6.68 × 10^4^ M^−1^, respectively. The limit of detection (LOD) toward Fe^3+^ of the PCaT and PCT was estimated to be 1.31 × 10^−6^ and 1.81 × 10^−6^ M, respectively, and the superior LOD of the PCaT was ascribed to its lowly crosslinked structure. The fluorescence of the solutions of these polymers that were quenched by Fe^3+^ ions recovered when trace CN^−^ anions were added because of the high stability constant of the CN^−^–Fe^3+^ complex. Micellar aggregates with a mean diameter of approximately 239.5 nm were formed by dissolving the PCaT in tetrahydrofuran (THF) solution. Our results suggest that the PCaT is a promising material for chemosensory applications.

## 1. Introduction

In recent decades, there has been remarkable growth in the use of conjugated polymers as fluorescent chemosensors for measuring pH, sensing chemical analytes, and detecting metal ions or biological species by employing their emission color or fluorescence intensity [1,2,3,4,5]. Fluorescent chemosensors generally contain a recognition site linked to a fluorophore, and a recognition event is translated into a fluorescent signal [6]. To date, a series of conjugated polymers have been synthesized to detect cations [7,8], anions [9,10], and pH [11,12] by incorporating specific receptors in the main or side chains of polymers, such as alkyl ethers [13], crown ethers [14], quinolones [15], bipyridines [16,17], and terpyridines [18,19]. A pyridine-based derivative, 2,2′:6′,2″-terpyridine (tpy), has an extremely high binding affinity toward many metal ions in low oxidation states because of its well-positioned ring of nitrogen atoms. Tpy derivatives are synthesized using Kröhnke-type condensation reactions of 2-acetylpyridine with corresponding aromatic aldehydes. Numerous studies have been conducted with tpy units used as recognition sites, and the marked effect that that choice of ligands has on the detection of metal ions in biological and environmental systems has been widely demonstrated [20,21].

Recent developments in controlled/living radical polymerization (CLRP) methods such as atom transfer radical polymerization [22,23], nitroxide-mediated radical polymerization [24], reversible addition-fragmentation chain transfer (RAFT) polymerization [25,26,27,28], reversible transfer-catalyzed polymerization [29], and single-electron-transfer-mediated living radical polymerization [30] offer new approaches to the preparation of block polymers with well-defined architectures, such as a simple experimental setup, a wide range of functional monomers, and complex macromolecular architectures with well-defined end groups of narrow polydispersity in the CLRP [31,32]. RAFT polymerization is a well-established CLRP method that has numerous appealing characteristics enabling control of the synthesis of a wide variety of macromolecular architectures and maintenance of molecular weight control, narrow molecular weight distributions, and functionality [33]. Mori et al. [34] reported the first successful synthesis of a carbazole-based side chain polymer (poly(*N*-vinylcarbazole)) using the RAFT technique. Most recently, Zhu et al. [35] prepared an amphiphilic copolymer poly(DBCzMA-*b*-NIPAM) through a two-step RAFT reaction using poly(6-(2,7-dibromo-9*H*-carbazole-9-yl) hexyl methacrylate) (poly(DBCzMA)) as the macro-chain transfer agent (macro-CTA) and poly(*N*-isopropylacrylamide) (PNIPAM) as the second monomer. Consequently, the modification of 2,7-dibromide groups in poly(DBCzMA-*b*-NIPAM) using the Suzuki coupling method (which reacted with a 4,4,5,5-tetramethyl-1,3,2-dioxaborolan-based carbazole monomer) obtained a poly(2,7-carbazole)-containing crosslinked polymer with the appearance of uniform core-shell fluorescent nanoparticles dissolved in water.

To the best of our knowledge, only a few studies on the synthesis of fluorescent chemosensor polymers using RAFT methods have been performed. Kanazawa et al. [36] synthesized a pH and temperature-responsive fluorescence polymer consisting of NIPAM and sulfamethazineacrylamide using RAFT polymerization, followed by the activation of terminal carboxyl groups with *N*-hydroxysuccinimide and reaction with 5-aminofluorescein. The fluorescence polymer exhibited a pH-dependent phase transition across the lower critical solution temperature, with specific intra-cellular uptake observed only under weak acidic conditions. In our previous study [37], we employed a two-step RAFT polymerization technique to synthesize a vinylcarbazole macro-CTA and a novel tpy-based block polymer, poly(*N*-vinylcarbazole)-*block*-poly[4’-((4-vinylphenyl) phenyl)-2,2′:6′,2″-terpyridine], to demonstrate the effect of a tpy unit on the sensory characteristics of fluorescent chemosensors. The final products exhibited a highly selective response to specific metal cations (Mn^2+^ and Ni^2+^).

In this study, we combined RAFT polymerization with a Heck coupling technique to synthesize a carbazole-functionalized crosslinked polymer (PCaT) with pendent tpy units as co-monomers in the Heck reaction. We examined the structural effect of the carbazole and tpy groups in the polymeric structure on the optical and chemosensory properties and self-assembly of the PCaT. These PCaT properties were also compared with those of a carbazole-tpy alternating conjugated polymer (PCT). Our contributions differ from those of previous studies in that the PCaT benefits from having a lowly crosslinked structure and luminescence properties, which result in a comparable sensing ability when mediated with metal ions. We discovered that the PCaT had a highly selective response and rapid, sensitive recognition to Fe^3+^, exhibiting a marked fluorescence change from bright blue to dark in the tetrahydrofuran (THF)–H_2_O mixture. The Stern–Volmer constant (*K*_sv_) and limit of detection (LOD) were 8.10 × 10^4^ M^−1^ and 1.31 × 10^−6^ M, respectively. The resultant PCaT–Fe^3+^ complex exhibited markedly selective fluorescence restoration with cyanide ions (CN^−^). In addition, the PCaT exhibited micellar aggregates in pure THF and in the THF–H_2_O mixture, which makes this polymer a promising material for high-potential chemosensory response and rapid microphase separation applications.

## 2. Experimental

### 2.1. Materials

**Scheme 1:**
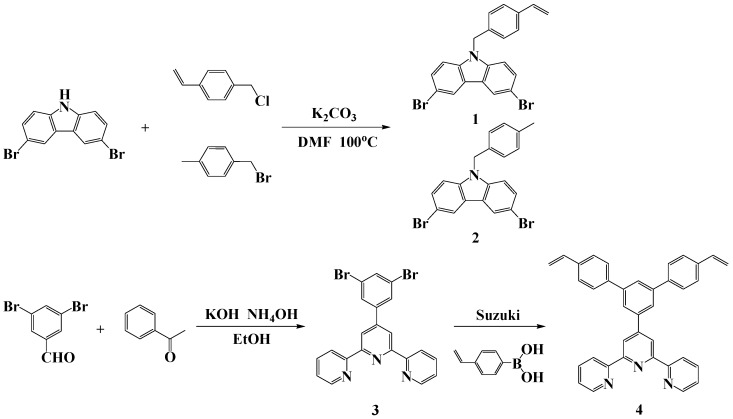
Synthetic routes of monomers.

**Scheme 2:**
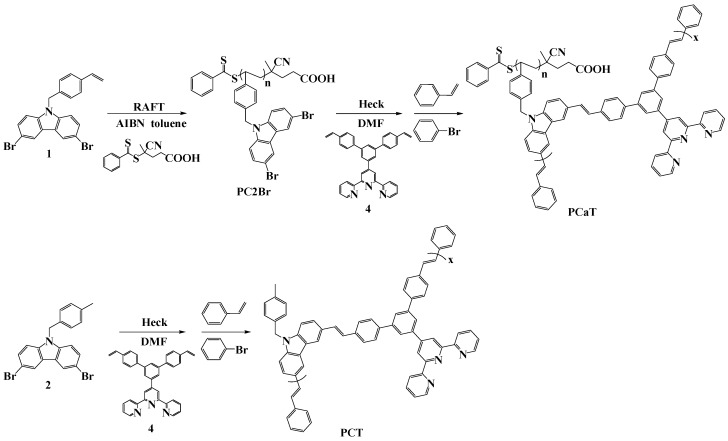
Synthetic routes of polymers (PCaT and PCT).

Synthetic routes for the carbazole- and terpyridine-based monomers and their corresponding polymers are shown in Scheme 1 and Scheme 2. 3,6-Dibromo-9*H*-carbazole and 4′-(3,5-bromophenyl)-2,2′:6′,2″-terpyridine (3) were synthesized following the processes reported previously [38,39,40]. The synthesis of 3,6-dibromo-9-(4-methylbenzyl)-9*H*-carbazole (2) and carbazole-tpy alternating conjugated polymer (PCT) is provided in the supporting information. Toluene was purified and distilled from sodium prior. *N*,*N*-dimethylformamide (DMF) was dried with appropriate drying agents, calcium hydride, or sodium, then distilled under reduced pressure and stored over 4 Å molecular sieves before use. 4-Vinylbenzyl chloride (99.0%, Acros, Geel, Belgium), 4-methylbenzyl bromide (97.0%, Aldrich, Saint Louis, MO, USA), 2-acetylpyridine (98.0%, Alfa, Ward Hill, MA, USA), 3,5-dibromobenzaldehyde (Alfa, 98.0%, Ward Hill, MA, USA), potassium carbonate (99.5%, Showa Chemical Industry Co., Ltd., Tokyo, Japan), 2,2′-azoisobutyronitrile (AIBN) (Aldrich, 98.0%, Saint Louis, MO, USA), 4-cyano-4-(thiobenzoylthio)pentanoic acid (97.0%, Aldrich, Saint Louis, MO, USA), tetrakis(triphenylphosphine) palladium (Pd(PPh_3_)_4_) (99.8%, Alfa, Ward Hill, MA, USA), palladium acetate (Pd(OAc)_2_) (98.0%, Aldrich, Saint Louis, MO, USA), tris(*o*-tolyl)phosphine (*p*(otol)_3_) (99.0%,TCI, Tokyo, Japan), and other reagents were purchased from Acros Chemical Co., Geel, Belgium and used without further purification.

### 2.2. Measurements

NMR spectra were recorded on a Bruker AMX-500 spectrometer (Billerica, MA, USA) with CDCl_3_ as a solvent, and chemical shifts (δ) were reported in ppm using tetramethylsilane (TMS) as an internal standard. Elemental analysis was performed on a Heraeus CHN-O rapid elemental analyzer. Fast Atom Bombardment mass spectra (FABMS) were obtained on a JEOL JMS-700 (Tokyo, Japan) mass spectrometer. Weight-average molecular weight (*M*_w_) and polydispersity index (PDI) of polymer were measured with a gel permeation chromatograph (GPC), model CR4A from Shimadzu, Kyoto, Japan using tetrahydrofuran (THF) as an eluent. The rate of elution was 1.0 mL/min; the instrument was calibrated with polystyrene standards (1000–136000 g/mol). Thermal analysis was performed using a differential scanning calorimeter (Perkin Elmer DSC 7, Waltham, MA, USA) at a scanning rate of 20 K/min under nitrogen atmosphere. Thermogravimetric analysis (TGA) was performed under nitrogen atmosphere at a heating rate of 20 K/min using a Perkin Elmer TGA-7 (Waltham, MA, USA) thermal analyzer. UV-vis absorption spectra were measured with a Jasco V-670 spectrophotometer. Fluorescence properties were detected with an OBB Quattro II fluorescence spectrophotometer. Fluorescence quantum yield (*Φ*_PL_) of polymers in solution were estimated at room temperature with poly(9,9-dihexylfluorene) (PF) as the standard (*Φ*_PL_ = 1.0). Dynamic light scattering (DLS) measurements were conducted with a Magic Droplet Model 100SB instrument (Sindatek Instrument Co., Ltd., Taipei, Taiwan) using a 4 mW He-Ne laser (λ = 632.8 nm) and equipped with a thermo stated sample chamber. Scanning electron microscopy (SEM) images were recorded using a JEOL JSM 5600 SEM system. Transmission electron microscopy (TEM) images were obtained through a JEOL TEM-2010 electron microscope with an accelerating voltage of 120 kV. Samples were prepared by drop-casting an aqueous solution of polymer on carbon-coated copper grids under ambient conditions.

### 2.3. Synthesis of Intermediates and Monomers (Scheme 1)

#### 2.3.1. Synthesis of 3,6-Dibromo-9-(4-vinylbenzyl)-9*H*-carbazole (1)

A mixture of 3,6-dibromo-9*H*-carbazole (3.25 g, 10.0 mmol), potassium carbonate (K_2_CO_3_) (2.76 g, 20.0 mmol), potassium iodide (1.0 mg, 0.006 mmol), and DMF (50 mL) was heated at 100 °C for 0.5 h, and then 4-vinylbenzyl chloride (2.30 g, 15 mmol) was added dropwise into the above system with stirring for 24 h. After cooling to room temperature, the mixture was poured into ice water, stirred, and then extracted with dichloromethane. The organic layer was successively washed with water twice, dried over anhydrous magnesium sulfate, and concentrated under reduced pressure. The crude product was purified by recrystallization from ethanol to afford 1 as a white solid (47.1%). ^1^H NMR (CDCl_3_, 500 MHz): δ_H_ (ppm) = 5.19–5.21 (dd, 1H, CH=C), 5.43 (s, 2H, aromatic, –CH_2_–), 5.62–5.69 (dd, 1H, CH=C), 6.56–6.66 (dd, 1H, CH=C), 6.87–7.22 (d, 4H, aromatic, Ar–H), 7.25–7.51 (d, 4H, aromatic, Ar–H), 8.16 (s, 2H, aromatic, Ar–H). Anal. Calcd. (%) for C_21_H_15_Br_2_N: C, 57.17; H, 3.43; N, 3.17. Found: C 57.62; H, 3.40; N, 3.12. FABMS (*m/z*) 439 M^+^.

#### 2.3.2. Synthesis of 4’-(3,5-Dibromophenyl)-2,2’:6’,2’’-terpyridine (3)

Yield: 45.0%. ^1^H NMR (CDCl_3_, 500 MHz): δ_H_ (ppm) = 7.35–7.37 (t, 2H, aromatic, Ar–H), 7.73 (s, 1H, aromatic, Ar–H), 7.86—7.89 (t, 2H, aromatic, Ar–H), 7.94 (s, 2H, aromatic, Ar–H), 8.65—8.66 (d, 4H, aromatic, Ar–H), 8.71–8.73 (s, 2H, aromatic, Ar–H). Anal. Calcd. (%) for C_21_H_13_Br_2_N_3_: C, 53.99; H, 2.80; N, 8.99. Found: C 54.12; H, 2.75; N, 8.87. FABMS (*m/z*) 465 M^+^.

#### 2.3.3. Synthesis of 4′-(4,4″-Divinyl-[1,1′:3′,1″-terphenyl]-5′-yl)-2,2′:6′,2″-terpyridine (4)

To a solution of 3 (0.47 g, 1.0 mmol), (4-vinylphenyl)boronic acid (0.33 g, 2.2 mmol), tetrakis(triphenylphosphine) palladium [Pd(PPh_3_)_4_] (0.046 g, 0.04 mmol) and aliquat 336 (0.02 g) in toluene (20 mL) was dissolved in 2 M aqueous potassium carbonate (K_2_CO_3_) solution (12 mL) and ethanol (10 mL). The solution was then stirred at 80 °C under nitrogen atmosphere. After stirring for 24 h, the solution was poured into an excess of methanol solution, and then the crude product was collected by filtration. After being dried, the crude product was dissolved in chloroform, washed with a saturated solution of EDTA, and purified via recrystallized from ethanol to afford 3 (91.0%). ^1^H NMR (CDCl_3_, 500 MHz): δ_H_ (ppm) = 5.30–5.32 (dd, 2H, CH=C), 5.82–5.86 (dd, 2H, CH=C), 6.77–6.83 (dd, 2H, CH=C), 7.35–7.38 (t, 2H, aromatic, Ar–H), 7.54–7.56 (d, 4H, aromatic, Ar–H), 7.71–7.73 (d, 4H, aromatic, Ar–H), 7.89–7.91 (m, 3H, aromatic, Ar–H), 8.06 (d, 2H, aromatic, Ar–H), 8.69–8.74 (d, 4H, aromatic, Ar–H), 8.83 (s, 2H, aromatic, Ar–H). Anal. Calcd. (%) for C_37_H_27_N_3_: C, 86.52; H, 5.30; N, 8.18. Found: C 86.68; H, 5.26; N, 8.23. FABMS (*m/z*) 513 M^+^.

### 2.4. Synthesis of Polymers (Scheme 2)

#### 2.4.1. Synthesis of Carbazole-Functionalized Polymer (PC2Br) Using the RAFT Method

A Schlenk tube was charged with monomer 1 (1.34 g, 3.04 mmol), 4-cyano-4-(thiobenzoylthio)pentanoic acid (CTP) (8.5 mg, 0.0304 mmol), AIBN (0.5 mg, 0.00304 mmol), toluene (7.6 mL), and a magnetic stirrer. The composition monomer/CTP/AIBN was used as 1000/10/1 in molar ratio. The mixture was purged with dry nitrogen and subjected to three freeze-pump-thaw cycles to remove any dissolved oxygen. Then the tube was sealed under vacuum and immersed in an oil bath for 48 h at 90 °C. The mixture was cooled to room temperature. After removal of the solvent, the residue was filtered in excess cold methanol to precipitate out the polymer. The resulting precipitate was placed in a Soxhlet apparatus and extracted with refluxed methanol for 72 h, following which it was dried in vacuum to give the product (18.4%). *T*_g_ = 186.6 °C, *T*_d5_ = 314.3 °C. *M*_w_ = 6.78 × 10^3^ g/mol, PDI = 1.12. ^1^H NMR (THF-*d*_8_, 500 MHz): δ_H_ (ppm) = 1.58 (s, CH_3_), 2.05 (s, –CH_2_–), 4.70–5.20 (br, –CH_2_–), 5.76–7.38 (br, aromatic, Ar–H), 7.99–8.25 (br, aromatic, Ar–H).

#### 2.4.2. Synthesis of Carbazole-Functionalized Copolymer (PCaT) Using the Heck Method

The carbazole-functionalized crosslinked polymer (PCaT) was prepared using palladium-catalyzed Heck coupling reactions of PC2Br/4. A mixture of PC2Br (50.0 mg), 4 (183.57 mg, 0.3577 mmol), Pd(OAc)_2_ (0.5 mg, 0.0022 mmol), *p*(otol)_3_ (2.9 mg, 0.011 mmol), trimethylamine (30 mg, 0.30 mmol), and DMF (3 mL) was carefully degassed. The mixture was stirred for 48 h at 100 °C under N_2_. Then, bromobenzene (0.08 g, 0.5 mmol) and styrene (0.052 g, 0.5 mmol) were added for the end capping by refluxing for 6 h each. The mixture was cooled to room temperature. After removal of the solvent, the residue was filtered in excess methanol to precipitate out the polymer. The resulting precipitate was placed in a Soxhlet apparatus and extracted with refluxed methanol for 48 h, after which it was dried in vacuum to give PCaT (10.3%). *T*_g_ = 198.3 °C, *T*_d5_ = 343.0 °C. *M*_w_ = 7.92 × 10^3^ g/mol, PDI = 1.14. ^1^H NMR (THF-*d*_8_, 500 MHz): δ_H_ (ppm) = 1.59 (s, CH_3_), 1.85 (s, –CH_2_–), 4.65–5.50 (br, –CH_2_–), 5.64–7.51 (br, aromatic, Ar–H), 8.02-8.32 (br, aromatic, Ar–H), 8.53–8.82 (br, aromatic, Ar–H).

### 2.5. Fluorescent Titration with Metal Ions

Fluorescent titration experiments were performed in THF solutions. Stock solutions (1.0 × 10^−3^ M) of the chloride or nitrate salts of Li^+^, Mg^2+^, Al^3+^, K^+^, Ca^2+^, Mn^2+^, Fe^3+^, Co^2+^, Ni^2+^, Zn^2+^, Ag^+^, Ba^2+^, and Pb^2+^ in deionized water were prepared. Titrations were performed by separately adding stock solutions to a test tube containing the polymer solution. All optical measurements were conducted immediately after the test solutions were prepared and thoroughly mixed. The final concentration of the polymer was 1.0 × 10^−6^ M. The percentage of water in THF was approximately 1.0%. The Stern–Volmer constant (*K*_sv_) was estimated according to following equation:

(*I*_o_/*I*) = 1 + *K*_sv_ [*Q*] (1)

where *I*_o_ and *I* are the intensities of the photoluminescence spectra without and with a quencher, respectively, *K*_sv_ is the Stern–Volmer constant (quenching coefficient), and [*Q*] is the concentration of the quencher ions. The stabilization of the polymer-Fe^3+^ complex (1.0 × 10^−6^ M in THF) was studied in the presence of some anions, including Cl^−^, Br^−^, I^−^, NO_3_^−^, NO_2_^−^, HSO_4_^−^, and CN^−^ (2.0 × 10^−5^ M). The limit of detection (LOD) of the polymer was calculated on the basis of fluorescence titrations. To obtain the slope, the fluorescence emission at maximal wavelength was plotted as a concentration of Fe^3+^ or CN^−^. The LOD was calculated using the following equation:

Limit of detection (LOD) = 3σ/*S* (2)

where σ is the standard deviation of the blank signal, and *S* is the slope between the fluorescence intensity versus [Fe^3+^] or [CN^−^].

### 2.6. Preparation of Micellar Aggregates

A suitable quantity of copolymer (PCaT) was first dissolved in anhydrous THF to form a homogeneous stock solution with a concentration of 0.5 mg mL^−1^. To obtain micellar aggregates in dispersions, deionized water (1 mL) was added to the THF solution (1 mL) at a rate of 500 μL h^−1^. Finally, spherical micelles were obtained by allowing the THF to evaporate under ambient conditions at room temperature.

## 3. Results and Discussion

### 3.1. Synthesis of Intermediates and Monomers

Scheme 1 outlines the synthetic route used to prepare the carbazole- and tpy-functionalized monomers (labeled 1 and 4, respectively, in the figure). The carbazole-functionalized monomer 1, 3,6-dibromo-9-(4-vinylbenzyl)-9*H*-carbazole, was prepared via a nucleophilic substitution reaction of 3,6-dibromo-9*H*-carbazole with 4-vinylbenzyl chloride at 100 °C. The crude product was purified through recrystallization from ethanol to afford 1, thus creating the monomer 1 at a yield of 47.1%. We then prepared 4′-(4,4″-divinyl-[1,1′:3′,1″-terphenyl]-5′-yl)-2,2′:6′,2″-terpyridine (4) in a 91.0% yield using a Suzuki coupling reaction between 4′-(3,5-dibromophenyl)-2,2′:6′,2″-terpyridine (3) and (4-vinylphenyl)boronic acid in the presence of potassium carbonate at 80 °C. The reaction was performed using Pd(PPh_3_)_4_ as the catalyst. The chemical structure and constitutional composition of the synthesized monomers were confirmed using proton nuclear magnetic resonance (^1^H NMR) spectroscopy. Figure S1 presents the ^1^H NMR spectra of monomers 1 and 4 and the corresponding structural assignments. The characteristic chemical shifts of *δ* = 8.16 (H_a_) and *δ* = 7.25–7.51 ppm (H_b_ and H_c_) were attributed to the carbazole singlet and doublet proton signals in monomer 1, respectively, whereas those of *δ* = 5.43, 6.56–6.66, 5.62–5.69, and 5.19–5.21 ppm were ascribed to aliphatic methylene (H_h_) and the vinyl group doublet of doublets (dd; H_f_, H_g_, and H_i_), respectively.

The ^1^H NMR spectrum of tpy-functionalized monomer 4 had peaks at approximately 8.69–8.83 ppm, which were assigned to shift to the ortho and meta positions labeled H_a_ and H_b_ of the pyridine unit, whereas the protons (H_c_ and H_g_) were the four aromatic protons of the 2,2′:6′,2″-terpyridyl segment at 8.06 and 7.35–7.38 ppm, respectively. The other aromatic protons corresponded to peaks at 7.54–7.91 ppm. The vinyl group dd (H_h_, H_i_, and H_j_) corresponded to peaks at 6.77–6.83, 5.82–5.86, and 5.30–5.32 ppm, respectively. Figure S2 displays the FABMS of the representative tpy-functionalized monomer 4. Additionally, the chemical structures of the synthesized monomers were confirmed using elemental analysis.

### 3.2. Synthesis and Thermal Properties of Polymers

The carbazole-based polymer (PC2Br) was synthesized using RAFT polymerization and was reacted in toluene at 90 °C using monomer 1 with a [M]_0_:[CTP]_0_:[AIBN]_0_ molar ratio of 1000:10:1. Table 1 presents a list of the RAFT polymerization results, and Scheme 2 displays the synthetic routes involved in the synthesis of PC2Br and PCaT. The structure of the PC2Br was characterized using GPC and ^1^H NMR spectroscopy. As indicated in Table 1, the number-average molecular weight (*M*_n_) and polydispersity index (PDI) of the PC2Br were 6.05 × 10^3^ g mol^−1^ and 1.12, respectively.

Figure S3a presents the ^1^H NMR spectrum of the PC2Br, which was recorded in THF-*d*_8_. The degree of polymerization (DP) of the PC2Br was calculated based on the integral ratio of protons H_a_ (at approximately 7.99–8.25 ppm) derived from the carbazole monomer, to methylene protons H_c_ in the CTP agent (at 2.05 ppm). The DP of the PC2Br was approximately 15.6, indicating that the moiety of the RAFT agent was attached to the ends of the PC2Br. Additionally, the *M*_n(NMR)_ of the PC2Br was 7162 g mol^−1^; this was calculated using the data in Figure S3a and the following equation:

*M*_n(NMR)_ = [(*H*_a_/*H*_c_) × *M*_n,carbazole_] + *M*_n,CTP_ (3)

where *H*_a_ is the integral of carbazole protons (–CH), *H*_c_ is the integral of methylene protons, *M*_n,carbazole_ is the molecular weight of monomer 1, and *M*_n,CTP_ is the molecular weight of CTP. The *M*_n_ determined using GPC was 6054 g mol^−1^. Consequently, the percentage of PC2Br chains end-capped with CTP groups was determined to be approximately 84.5%.

The PCaT was successfully synthesized from PC2Br and tpy-based monomer 4 through a palladium-mediated Heck coupling reaction (Scheme 2). Figure S3b presents the ^1^H NMR spectrum of the PCaT in THF-*d*_8_. The molar ratio of carbazole to tpy units was calculated to be 15.6:1 by comparing the integrated peak areas of carbazole protons (H_c_: 4.65–5.50 ppm) to those of terpyridyl protons (H_a_: 8.53–8.82 ppm) in monomer 4. We attributed the low tpy ratio to the steric effects and stable resonance of carbazole units in the PC2Br, which reduced the polymerization reactivity of the tpy monomer. The decomposition temperatures (*T*_d_s) of PC2Br and PCT at a 5% weight loss were observed between 314.3–343.0 °C. The residual weights of the PC2Br and PCT at 800 °C were above 24.8 and 40.1%, respectively, indicating that introducing tpy units enhances thermal stability (Figure S4). The PCT was prepared using 3,6-dibromo-9-(4-methylbenzyl)-9*H*-carbazole (2) and monomer 4 at a molar ratio of 1:1.1. The molar percentage of monomer 4 in the PCT was estimated to be approximately 45.0% using the integrated peak areas of the methylene protons (5.43 ppm) of carbazole and the 2,2′:6′,2″-terpyridyl segment at 8.63–8.85 ppm of monomer 4. Moreover, the PCaT and PCT had glass transition temperatures (*T*_g_) of approximately 198.3 and 159.8 °C, respectively. This finding suggests that incorporating lowly crosslinked structures along the PCaT copolymer backbones provides high rigidity, leading to high *T*_g_.

### 3.3. Optical Properties of Polymers

**Figure 1:**
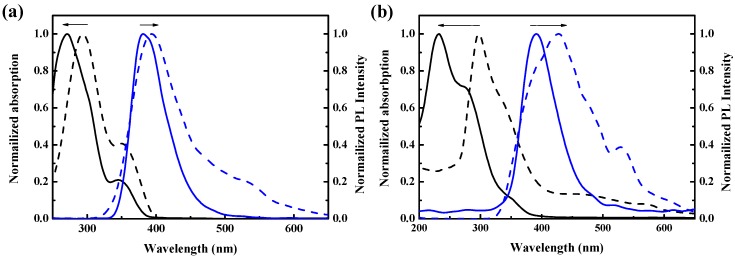
Absorption and photoluminescence spectra of (**a**) PCaT and (**b**) PCT in THF solution (solid line) and in thin film (dotted line).

Figure 1 displays the absorption and photoluminescence spectra of the PCaT and PCT in THF and as thin films spin-coated from the THF solution (10 mg/mL). The corresponding data is summarized in Table 2. In THF, the PCaT showed strong absorption that peaked at 271 nm, resulting from π-π* electronic transitions of the carbazole units. A weak transition shoulder in the PCaT spectrum was observed at approximately 346 nm, which was attributed to intramolecular charge transfer (ICT) between the carbazole donor groups and tpy acceptors, leading to the observed ICT interaction and a bathochromic shift [41,42]. By contrast, the PCT spectrum had absorption bands at 232 and 275 nm. In the spectra of the thin films, the absorption maximum of the PCaT and PCT was red-shifted by approximately 22 and 65 nm relative to the solution state spectra, respectively, resulting from aggregation caused by intra- or inter-chain interactions. These results suggest that an increase in polymer chain planarization for the PCT facilitates aggregation, which causes a large red-shift of the absorbance in the solid state.

In the fluorescence spectra, no emission peaks were observed in the PC2Br, whereas emission peaks derived from PCaT were observed at 381 and 394 nm for PCaT in THF and as a thin film, respectively. Given the ^1^H NMR characterization and optical properties, the formation of the PCaT was expected. However, the emission peaks of the PCT were situated at 391 and 428 nm for the PCaT in THF and as a thin film, respectively, implying that excimers or exciplexes formed in the thin film. The Stokes shifts of the PCaT and PCT were 110 and 159 nm, respectively (Table 2). This difference was due to the donor-acceptor interactions that occurred between the polymer backbone and tpy acceptor group. The large shift may also result from structural differences between the ground and excited states in addition to migrated excitons in the segments of the chain, allowing the ring to rotate more flexibly. The fluorescence quantum yield (*Φ*_PL_) of the PCaT and PCT was estimated using poly(9,9-dihexylfluorene) as a reference (*Φ*_PL_ = 1.0). The yields were 6.2 × 10^−2^ and 5.1 × 10^−1^, respectively, which suggest that incorporating a low ratio of tpy units into the carbazole backbones for the PCaT reduced coplanar conformations, thereby causing ineffective conjugation lengths to arise from orbital interactions between the carbazole and tpy groups.

### 3.4. Ion Sensing Properties

**Figure 2:**
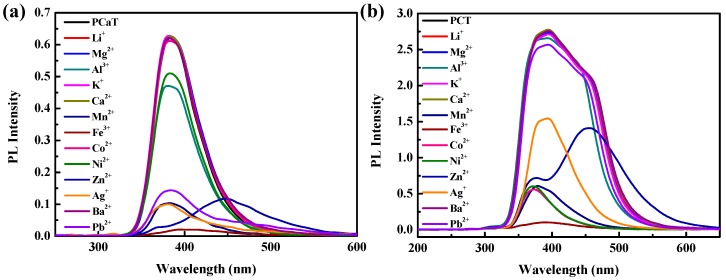
Photoluminescence spectra of (**a**) PCaT and (**b**) PCT in the presence of various metal ions (excitation: 235 nm). Concentration of polymers: 1.0 × 10^−6^ M in THF, concentration of metal ions: 5.0 × 10^−5^ M in water.

Tpy groups are generally employed as ligands to combine metal cations for the generation of complexes. Figure 2 illustrates the effect of complexation with various metal ions (Li^+^, Mg^2+^, Al^3+^, K^+^, Ca^2+^, Mn^2+^, Fe^3+^, Co^2+^, Ni^2+^, Zn^2+^, Ag^+^, Ba^2+^, and Pb^2+^) on the fluorescence spectra of the PCaT and PCT in the THF‒H_2_O solution. When the polymer solution was titrated separately with Mn^2+^, Fe^3+^, Ni^2+^, Zn^2+^, Ag^+^, or Pb^2+^, the photoluminescence intensities decreased significantly, indicating that the tpy chelating moiety in the polymer chain effectively transferred energy from the conjugated polymer backbone to these cations, thus leading to the quenching of the fluorescence of the PCaT and PCT. Notably, the fluorescence of both polymers was completely quenched by Fe^3+^ ions at an ion concentration of 1.0 × 10^−4^ M, indicating their high selectivity toward this cation. The quenching mechanism can be attributed to the photoinduced energy transfer (PET) from the polymer chain, with the initial PET occurring during a collision (dynamic quenching) between the excited fluorophore and a metal ion. Thus, the PCaT and PCT could be used as polymer chemosensors for sensing cations.

**Figure 3:**
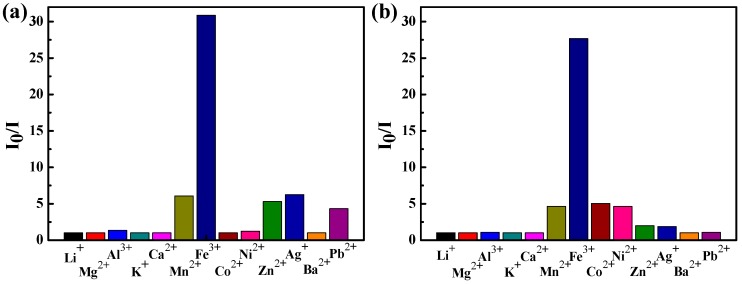
Photoluminescence response profiles for (**a**) PCaT and (**b**) PCT solutions by adding various cations in THF.

**Figure 4:**
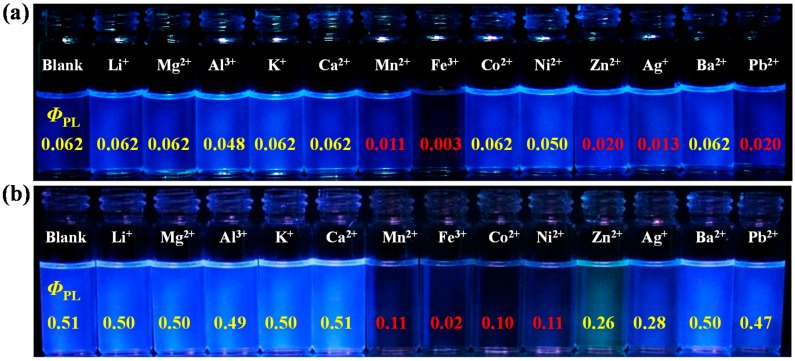
Fluorescence colors of (**a**) PCaT and (**b**) PCT solutions after the addition of various cations. The values in images are their corresponding quantum yields (*Φ*_PL_s) after adding the given cations.

Figure 3 displays the photoluminescence response profiles (i.e., *I*_o_/*I*) of the polymers in the presence of various metal ions at an ion concentration of 1.0 × 10^−4^ M. The emission colors of fluorescence response to various metal ions were visibly different (Figure 4). We discovered that the PCaT and PCT had a high sensitivity toward Fe^3+^, with a clear fluorescence change from bright blue to dark in the THF‒H_2_O mixture. These results indicated that the shorter diameter (1.28 Å) and higher charge (1.83) of Fe^3+^ might lead to a higher electron-accepting capability and, consequently, more stable complexes. Moreover, these two factors (i.e., diameter and charge) may play a crucial role in determining the coordination strength of Fe^3+^ ions accompanying tpy units [43]. The five electrons (Fe^3+^: *d*^5^ electron configuration) may be present as two orbitals occupied by pairs of electrons and one orbital with a single occupancy, leading to an inner-orbital complex (which is more stable than other host‒metal complexes) [44]. The *Φ*_PL_ of the PCaT in the presence of Fe^3+^ ions was approximately 95.2% lower than when Fe^3+^ ions were not present (3.0 × 10^−3^ vs. 6.2 × 10^−2^); by contrast, the *Φ*_PL_ for the PCT in the presence of Fe^3+^ ions was decreased by approximately 96.1% (from 5.1 × 10^−1^ to 2.0 × 10^−2^). Moreover, adding Zn^2+^ ions in PCaT and PCT solutions caused a large red-shifted emission peak by approximately 66 and 65 nm relative to the solution state spectra, respectively, which was attributed to the ICT processes. The cation receptor may have played the role of an electron receptor, facilitating the enhancement of the receptor-cation interactions. Subsequently, a red shift in emission would be observed [45].

**Figure 5:**
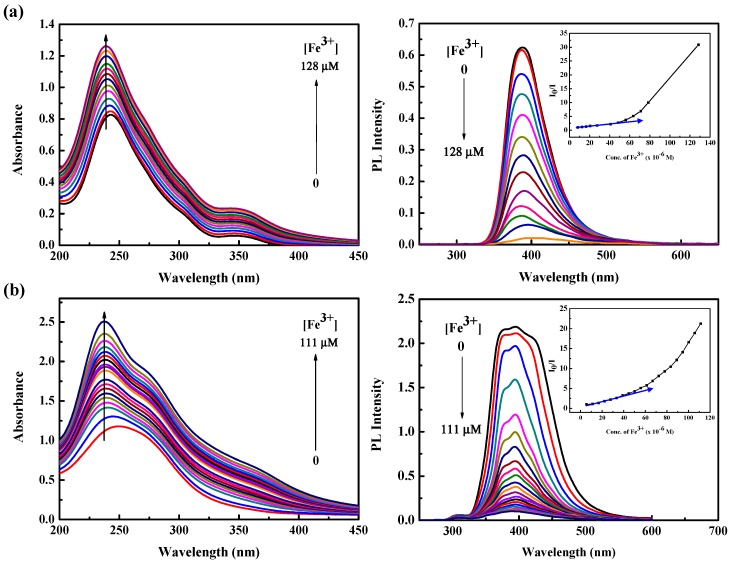
Absorption (left) and photoluminescence (right) spectral variations of (**a**) PCaT and (**b**) PCT in the presence of various concentrations of Fe^3+^. Concentration of polymers: 1.0 × 10^−6^ M in THF; Inset: Stern‒Volmer plot of fluorescence quenching with various concentrations of Fe^3+^ ion.

Figure 5 displays the absorption spectral variations of PCaT and PCT upon the addition of Fe^3+^ at a concentration of approximately 1.28 × 10^−4^ or 1.11 × 10^−4^ M. When the addition of Fe^3+^ was higher, both polymers had absorption spectra with higher intensities, which resulted from complexation between the 2,2′:6′,2″-terpyridine units and the positively charged Fe^3+^. To quantitatively evaluate the fluorescence quenching achieved by adding Fe^3+^, the fluorescence spectra and response of polymers to titration with Fe^3+^ were studied in THF. The photoluminescence intensities decreased with increasing Fe^3+^ concentration (Figure 5). Additionally, the slope of the Stern‒Volmer plot increased noticeably when Fe^3+^ concentration was greater than 6.0 × 10^−5^ M (i.e., static quenching), resulting from strong tpy chelation to transition metal ions. *K*_sv_ for the PCaT and PCT in the presence of low Fe^3+^ concentration (less than approximately 5.0 × 10^−5^ M) was 8.10 × 10^4^ and 6.68 × 10^4^ M^−1^, respectively. The estimated LOD for the PCaT and PCT toward Fe^3+^ was as low as 1.31 × 10^−6^ and 1.81 × 10^−6^ M, enabling both polymers to be used as selective and sensitive Fe^3+^ probes. The standard deviation (σ) of the PCaT and PCT was 3.59 × 10^−3^ and 4.85 × 10^−3^ M, respectively. The synthesized polymers indicated the presence of Fe^3+^ ions based on fluorescence “turn-off” characteristics that resulted from efficient energy transfer between the polymer backbone and tpy moieties.

**Figure 6:**
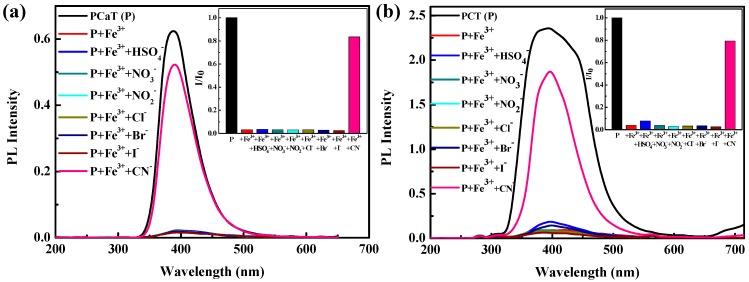
Photoluminescence spectra of (**a**) PCaT‒Fe^3+^ and (**b**) PCT‒Fe^3+^ in the presence of various anions. Inset: photoluminescence response profiles of polymer‒Fe^3+^ in the presence of various anions.

**Figure 7:**
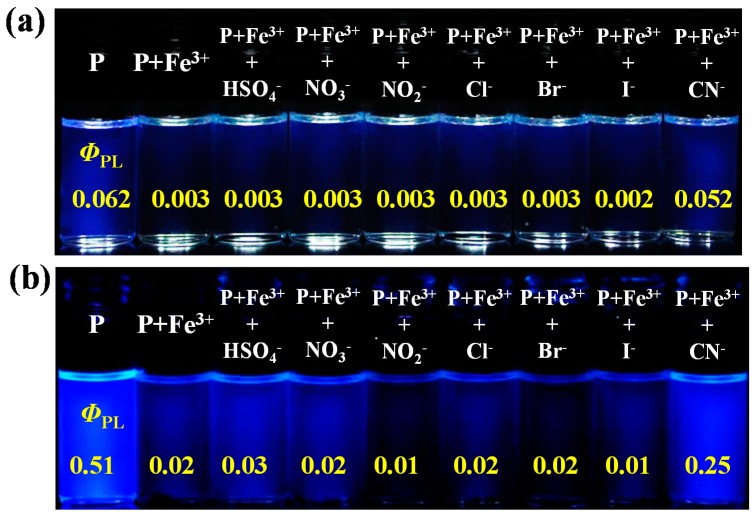
Fluorescence colors of (**a**) PCaT‒Fe^3+^ and (**b**) PCT‒Fe^3+^ solutions after the addition of various anions. The values in images are their corresponding quantum yields (*Φ*_PL_s) after adding the given anions.

To determine whether the polymer−Fe^3+^ complex can be used as an anion selective probe, the response of the complex toward anions (Cl^−^, Br^−^, I^−^, NO_3_^−^, NO_2_^−^, HSO_4_^−^, and CN^−^) was investigated (Figure 6). The fluorescence response profiles (i.e., *I*/*I*_o_) of the polymer−Fe^3+^ complexes in the presence of anions with a concentration of 2.0 × 10^−5^ M are also illustrated in the insets of Figure 6. These data demonstrate that the fluorescence intensity of the polymer−Fe^3+^ complex increased considerably upon the addition of CN^−^ ions, whereas complexation with other anions induced no obvious shift in the emission peak because of the poor coordination of Fe^3+^ with these anions. This finding is attributed to the high stability of the Fe^3+^–CN^−^ complex [46]. The PCaT–Fe^3+^ could be used for the sensitive and selective detection of CN^−^ ions with a linear range from 1.0 × 10^−5^ M to 6.0 × 10^−4^ M and an LOD of 5.62 × 10^−6^ M, whereas the linear range and LOD of PCT–Fe^3+^ was estimated to be 1.0 × 10^−5^–6.5 × 10^−4^ M and 8.27 × 10^−6^ M, respectively. Hence, the fluorescence intensities of the PCaT and PCT were revived by the transformation from a polymer–Fe^3+^ complex (quenched) into a free polymer (revived). As illustrated in Figure 6, the fluorescence intensity recovered up to 83.4% and 79.3% of the original intensity for the PCaT and PCT, respectively. The *Φ*_PL_ of the PCaT–Fe^3+^ in the presence of CN^−^ ions was restored to 79.0% (from 3.0 × 10^−3^ to 5.2 × 10^−2^), whereas the *Φ*_PL_ for the PCT in the presence of CN^−^ ions was restored to 45.1% (from 2.0 × 10^−2^ to 2.5 × 10^−1^), enabling the polymers to be used as sensitive CN^−^ sensors (Figure 7).

### 3.5. Self-Assembly of Polymer in THF Solution

**Figure 8:**
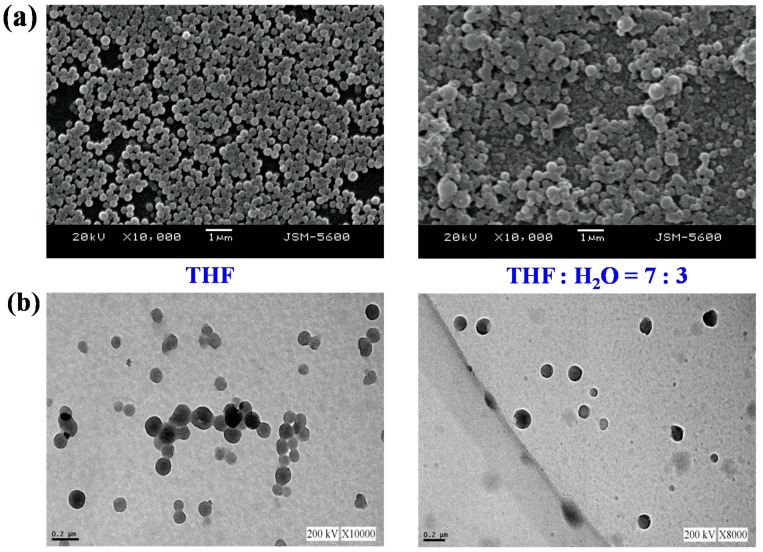
(**a**) SEM and (**b**) TEM images of spherical micelles formed from PCaT in different amounts of THF‒H_2_O. The polymer concentration was 0.5 and 0.25 mg mL^−1^ for SEM and TEM observation, respectively.

We prepared micellar aggregates by dissolving the PCaT in THF and gradually adding water to dilute the homogeneous PCaT solution. Figure 8 presents SEM and TEM images of the micellar aggregates obtained using the PCaT (0.5 mg mL^−1^) in the THF‒H_2_O mixtures with varying water content. We also observed the morphological aggregates of the PCaT dissolved in the pure THF solution. Specifically, using SEM observation (Figure 8a), it was observed that the polymer had spherical micelle diameters of approximately 200–250 nm, and the aggregates were attributable to the relatively short hydrophobic tpy units (at approximately 6.0%) in the polymer core [47]. Notably, the PCaT, which has long polycarbazole segments, was employed for the preparation of the crosslinked polymer, resulting in the formation of spherical micelles with thick polycarbazole shells. However, we observed no micellar aggregates of the PCT dissolved in the pure THF solution, indicating that THF was an appropriate solvent for both copolymer units (i.e., carbazole and tpy monomers) and that the PCT could be adequately dissolved in THF without aggregation occurring. Figure S5 displays the energy dispersive spectroscopy (EDS) data of PCaT–Fe^3+^ in THF.

When the volume of THF:H_2_O was 9:1 and 7:3, the diameter of the spherical micelles of the PCaT was in the range of approximately 200–300 and 300–500 nm, respectively. These results indicate that, with the gradual addition of water to the PCaT solution in THF, the solubility of the solvent progressively worsened for the tpy units; after the addition of a critical amount of water, the polymer solutions exhibited a microphase separation. The micelles further worsened and became increasingly irregular after the water content was increased to 50 vol % (i.e., the volume of THF:H_2_O was 5:5). When the water content was greater than 70 vol %, no regular micellar aggregates formed, despite there being fewer spherical micelles. These results indicate that increasing the water content leads to the disruption and dissociation of micellar aggregates into small micelles because of the insoluble states of the two copolymer units at high water concentrations, resulting in the collapse of the micellar aggregates. Figure S6 illustrates the DLS distribution of spherical micelles formed from the PCaT in the pure THF solution, with the average micelle diameter being approximately 239.5 nm. This finding revealed that the morphological aggregates of the PCaT depend on both copolymer composition and preparation conditions, such as the copolymer length, copolymer ratio, molecular interaction, hydrophobic and hydrophilic properties of the copolymer, and solvent properties [48]. TEM was performed to confirm this observation of the spherical micelles. Figure 8b presents a TEM image of the micelles when the water content was 0 and 30 vol %, respectively, and the image is consistent with the SEM observation. These results demonstrate that this process is an efficient crosslinking approach for the preparation of uniform micelles.

## 4. Conclusions

A new carbazole-functionalized crosslinked polymer, PCaT, with tpy groups as receptors was synthesized, and its corresponding optical and sensory characteristics were evaluated and compared with those of a PCT. The PCaT exhibited high selectivity toward Fe^3+^ ions (“turn-off”) with a Stern‒Volmer constant (*K*_sv_) of 8.10 × 10^4^ M^−1^ and an LOD of 1.31 × 10^−6^ M. The superior LOD sensing of the PCaT was attributed to the incorporation of tpy units crosslinked into the structure of the polymer. Furthermore, the polymer‒Fe^3+^ complex demonstrated particularly selective fluorescence restoration (“turn-on”) abilities toward CN^-^ ions. Micellar aggregates formed from the PCaT in pure THF and in different amounts of THF‒H_2_O (i.e., when the volume of THF:H_2_O was 9:1 and 7:3), as revealed in SEM and TEM images of the copolymer. These results indicate that the PCaT copolymer micelles have high potential for use in environmental applications, meet the selection requirements for detecting Fe^3+^ ions, and exhibit self-assembly properties.

## Figures and Tables

**Table 1:** Molecular weights and thermal properties of polymers.

Polymer	Yield (%)	*M*_w_ (×10^3^) ^a^	PDI ^a^	*T*_g_ (°C) ^b^	*T*_d_ (°C) ^c^	Molar ratio (*x*:*y*) ^d^
PC2Br	18.4	6.78	1.12	186.6	314.3	―
PCaT	10.3	7.92	1.14	198.3	343.0	94:6
PCT	35.6	12.2	1.65	159.8	268.7	55:45

^a^ *M*_w_ and polydispersity index (PDI) of the polymer were determined by gel permeation chromatography using polystyrene standards in tetrahydrofuran. ^b^ Glass transition temperatures by differential scanning calorimeter under N_2_ at a heating rate of 20 °C/min. ^c^ Temperatures at 5% weight loss. ^d^ Final molar ratio of carbazole and terpyridine-based monomer was determined by ^1^H NMR spectra.

**Table 2:** Optical properties of polymers.

Polymer	UV-vis λ_max_ sol’n (nm) ^a^	UV-vis λ_max_ film (nm) ^a^	PL λ_max_ sol’n (nm) ^b^	PL λ_max_ film (nm) ^b^	Stokes shift ^c^	*Φ*_PL_^d^
PCaT	271, 346s	293, 350s	381	394	110	0.062
PCT	232, 275s	297	391	428, 529s	159	0.51

^a^ Measured in THF solution (1.0 × 10^−6^ M). Superscript s means the wavelength of the shoulder. ^b^ The excitation wavelength was 235 nm in THF for polymers. ^c^ Stokes shift = PL_(film)_/nm − UV_(film)_/nm. ^d^ These values of quantum yield were measured using poly(9,9-dihexylfluorene) as a standard (1.0 × 10^−7^ M, assuming a quantum yield of unity).

## Supplementary Materials

The following are available online at www.mdpi.com/2073-4360/9/9/427/s1. Figure S1: ^1^H NMR spectra of monomers (a) 1 and (b) 3. Figure S2: FABMS of monomer 4. Figure S3: ^1^H NMR spectra of polymers (a) PC2Br and (b) PCaT. Figure S4: TGA curves of PC2Br and PCaT. Figure S5: Energy dispersive spectroscopy (EDS) data of polymer PCaT-Fe^3+^ in THF. Figure S6: Dynamic light scattering (DLS) measurement of PCaT in THF.
