# Supplementary material for: Synthesis, Chemosensory Properties, and Self-Assembly of Terpyridine-Containing Conjugated Polycarbazole through RAFT Polymerization and Heck Coupling Reaction
Source: Polymers (Basel). 2017 Sep 7;9(9):427. doi: 10.3390/polym9090427 (PMC6418694; doi:10.3390/polym9090427)
Supplement: Supplementary file 1 [file polymers-09-00427-s001.pdf]

polymers

MDPI

Supporting information for

Synthesis, Chemosensory properties, and Self-Assembly of Terpyridine-Containing Conjugated Polycarbazole through RAFT Polymerization and Heck Coupling Reaction

Po-Chih Yang*, Si-Qiao Li, Yueh-Han Chien, Ta-Lun Tao, Ruo-Yun Huang and Hsueh-Yu Chen

Department of Chemical Engineering and Materials Science, Yuan Ze University, Chung-Li, Taoyuan City 32003, Taiwan
E-mail address: pcyang@saturn.yzu.edu.tw (P. C. Yang).
Tel: +886 3 4638800 Ext. 3556, Fax: +886 3 4559373

Synthesis of 3,6-Dibromo-9-(4-methylbenzyl)-9*H*-carbazole (2)

3,6-Dibromo-9-(4-methylbenzyl)-9*H*-carbazole (2) was prepared by a procedure similar to that for 1, using 1-(bromomethyl)-4-methylbenzene instead of 4-vinylbenzyl chloride. Yield: 65.6%. ^{1}H NMR (acetone-d_6, 500 MHz): δ_H (ppm) = 2.22 (s, 1H, -CH_3), 5.61 (s, 2H, -CH_2-), 7.05 (d, 4H, aromatic, Ar-H), 7.55 (d, 4H, aromatic, Ar-H), 8.39 (s, 2H, aromatic, Ar-H). Anal. Calcd. (%) for $C_{20}H_{15}Br_2N$: C, 55.97; H, 3.52; N, 3.26. Found: C 56.06; H, 3.48; N, 3.30.

Synthesis of Carbazole-functionalized alternating conjugated polymer (PCT)

A mixture of 3,6-dibromo-9-(4-methylbenzyl)-9*H*-carbazole (2) (0.21 g, 0.50 mmol), 3 (0.28 g, 0.55 mmol), Pd(OAc)$_2$ (2.2 mg, 0.01 mmol), *p*(otol)$_3$ (13.1 mg, 0.05 mmol), trimethylamine (136.6 mg, 1.35 mmol) and DMF (3 mL) was carefully degassed. The mixture was stirred for 48 h at 100 °C under N_2. Then, bromobenzene (0.08 g, 0.5 mmol) and styrene (0.052 g, 0.5 mmol) were added for the end capping by refluxing subsequently for 6 h each. The mixture was cooled to room temperature. After removal of the solvent, the residue was filtered in excess methanol to precipitate out the polymer. The resulting precipitate was placed in a Soxhlet apparatus and extracted with refluxed methanol for 48 h and then was dried in vacuum to give PCT (35.6%). T_g = 159.8 °C, T_{d5} = 268.7 °C. M_w = 1.22 × 10^4 g/mol, PDI = 1.65. ^{1}H NMR ($CDCl_3$, 500 MHz): δ_H (ppm) = 2.25-2.30 (br, -CH_3), 5.43 (br, -CH_2-), 6.95-7.90 (br, aromatic, Ar-H), 8.14-8.18 (br, aromatic, Ar-H), 8.63-8.85 (br, aromatic, Ar-H).

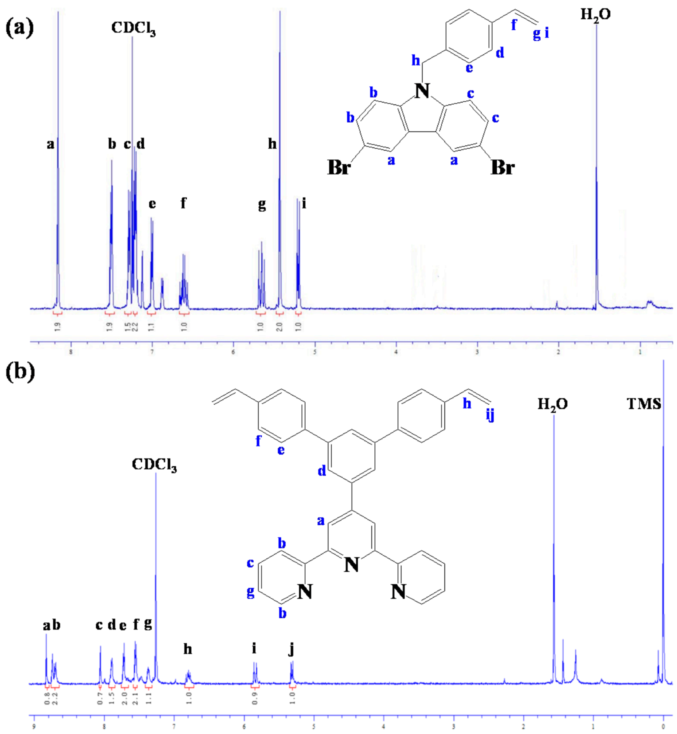

Figure S1. ^{1}H NMR spectra of monomers (a) 1 and (b) 3.

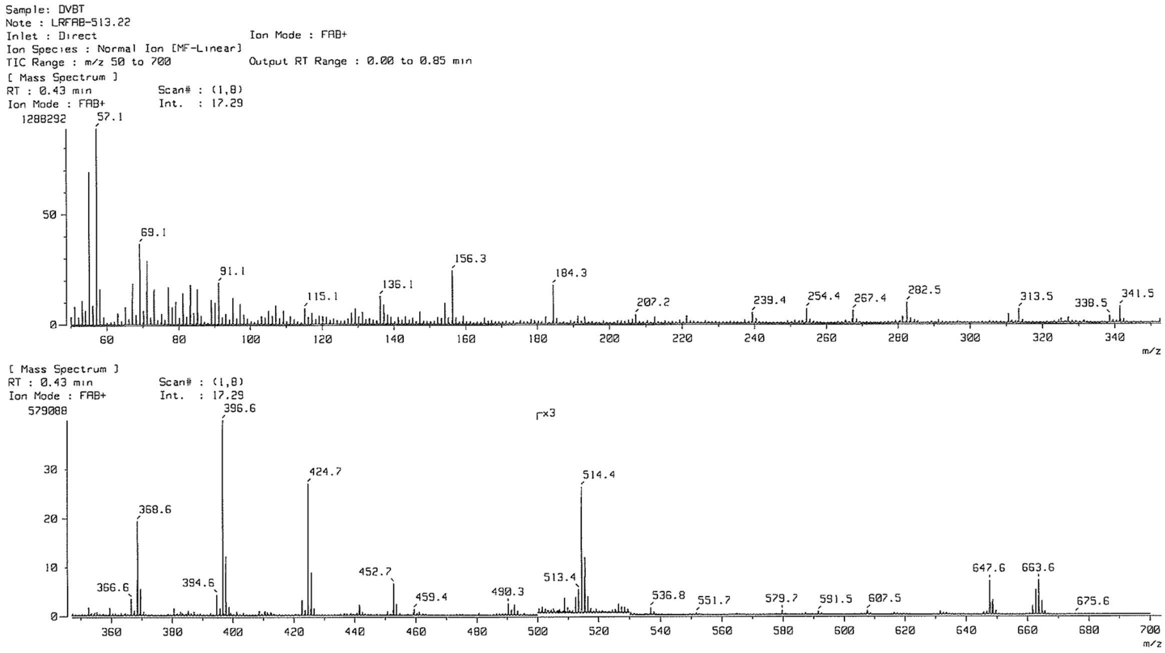

Figure S2. FABMS of monomer 4.

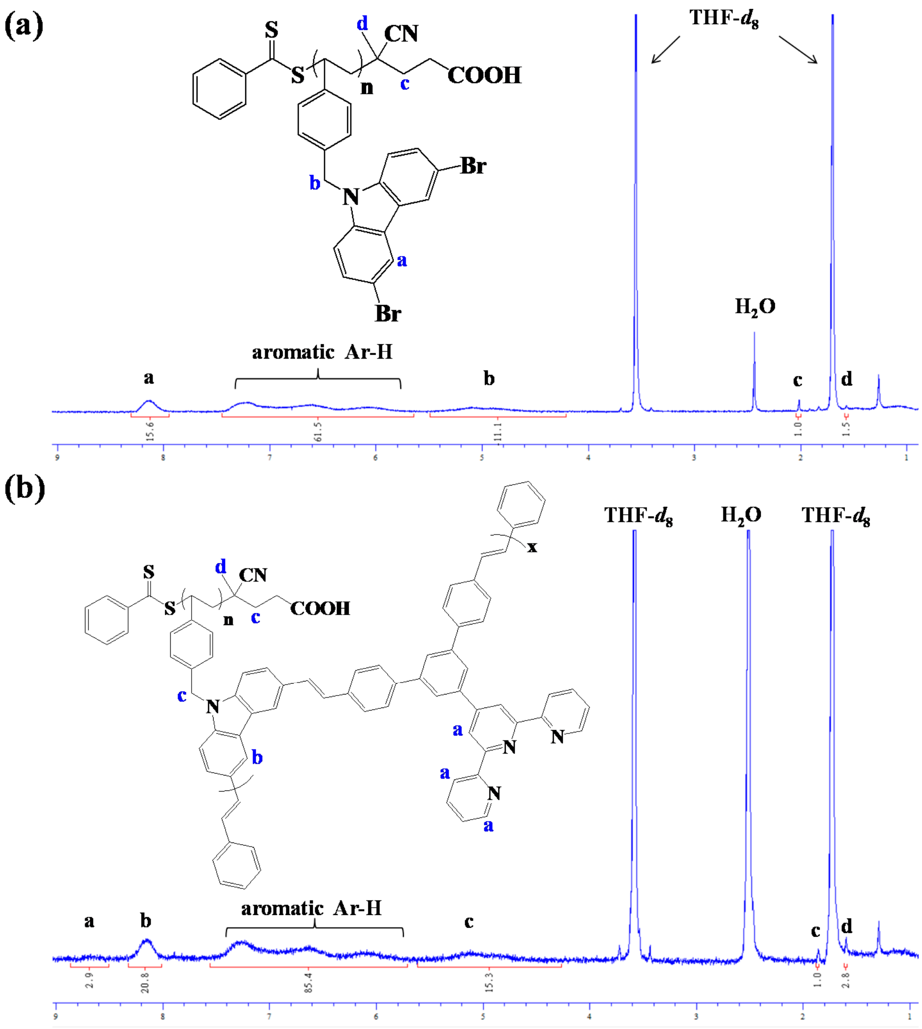

Figure S3. ^{1}H NMR spectra of polymers (a) PC2Br and (b) PCaT.

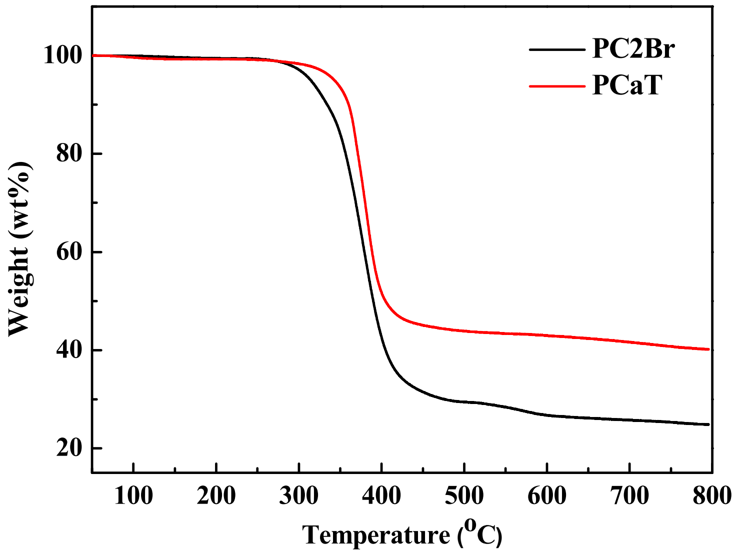

Figure S4. TGA curves of PC2Br and PCaT.

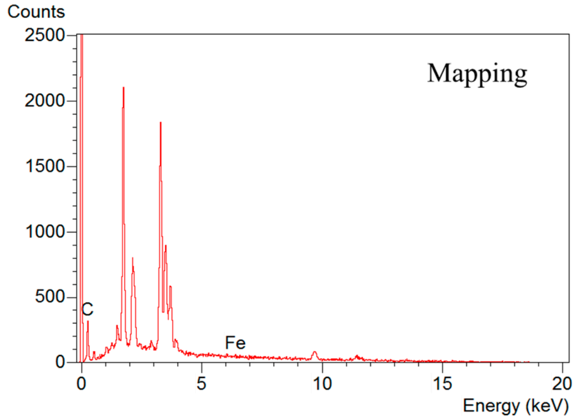

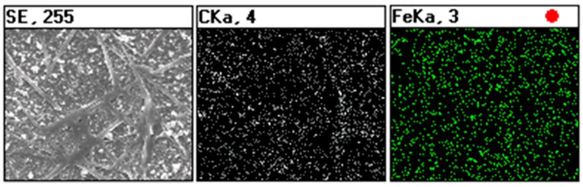

Element	Spect. Type	Inten. Corrn.	Std Corrn.	Element (%)	Sigma (%)	Atomic (%)
C K	ED	1.982	1.12	97.11	3.76	99.36
Fe K	ED	0.890	1.10	2.89	3.76	0.64
Total				100.00		100.00

Figure S5. Energy dispersive spectroscopy (EDS) data of polymer PCaT–Fe^{3+} in THF.

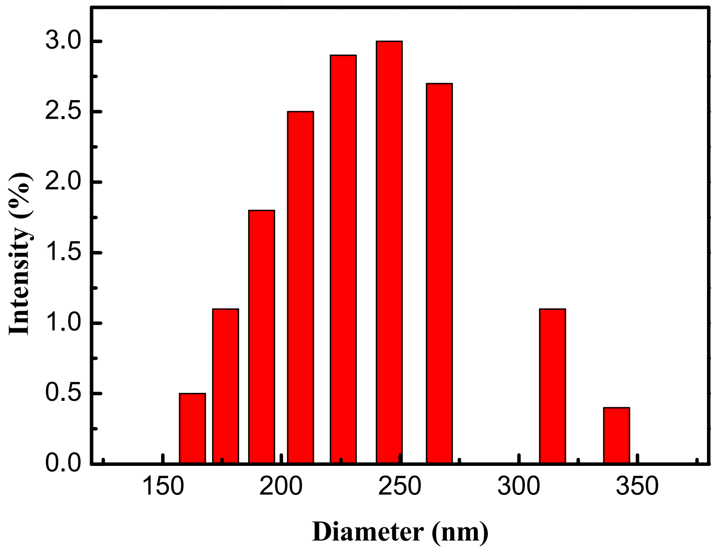

Figure S6. Dynamic light scattering (DLS) measurement of PCaT in THF.
